# Reshaping the global health agenda: female genital cutting

**DOI:** 10.3402/meo.v21.31023

**Published:** 2016-06-07

**Authors:** Nina Al-Saadi, Harun Khan, Sameera Auckburally, Adam Al-Saadi, Tasnia Khan

**Affiliations:** 1 Imperial College London, London, United Kingdom; 2 St George’s University of London, London, United Kingdom

Female genital cutting (FGC) is described by the World Health Organization as ‘all procedures that involve partial or total removal of the female external genitalia or other injury to the female genital organs for non-medical reasons’ (1). Its practice is common in at least 29 countries across Africa and the Middle East (2) – currently affecting 125 million girls and women worldwide. Due to globalization, specifically increasing trends in migration, instances of FGC are increasingly common in the developed world. For example, 137,000 girls residing in the UK in 2011 were found to have undergone FGC (3). Despite this, the increasing trend of FGC in the developed world has not been uniformly met with suitable mechanisms to support these patients.

With increased societal stigma and grave misinformation, funding for FGC support structures has largely ignored sensitive issues surrounding FGC, for example, the social and economic structures that propagate abuse against women, such as poverty (4). Without promoting dialogue and tackling FGC on a broader level, any progress we make to tackle FGC will be stunted. In this article, we aim to briefly summarise methods to tackle FGC and underline any improvements that can help better support those at risk or who have undergone FGC.

## Health education

FGC exemplifies a severe form of discrimination against women, stemming from historical inequalities between the sexes (1). Although globally regarded as a human rights violation, FGC continues to be practised due to social convention (5). Alongside this, FGC can have detrimental effects on the individual – through its associated immediate and long-term health problems ranging from physical to psychological impact (6). Studies have found that severe pain, haemorrhage, shock, dysuria, and death are amongst the most common immediate complications (6). Infections, including contracting human immunodeficiency virus, and psychological trauma can be classified as both immediate and long-term complications of FGC. Additional long-term complications include infertility and an increased risk of cervical cancer (7, 8). If fertility is maintained, the damage to the genital organs through FGC can pose a threat to both the foetus and mother during childbirth (6).

Despite the obvious health implications of FGC, awareness of these issues is poor. A recent case, which clearly demonstrated the lack of awareness of FGC in British medical practice, led to the prosecution of an obstetrician in the UK (9). This obstetrics registrar placed a single continuous suture on a patient who was subjected to FGC, as a child, in order to stop his patient bleeding post-partum. The suture technique used by the registrar was said to be a form of FGC known as re-infibulation. The doctor had never received any formal teaching on FGC – despite his experiences in obstetrics and gynaecology. This case truly stresses the importance of incorporating FGC awareness in society and, in particular, amongst our healthcare professionals. The awareness of FGC is low, but there is potential for better platforms for this issue. At the BMA medical student conference in 2014, delegates voted in favour of doctors being ‘aware of the short and long-term effects of FGC through comprehensive medical school teaching’. FGC teaching was felt to be an important, yet missing, part of their current medical education (10). In this way, increasing education targeting healthcare professionals can help us strengthen health systems globally to better support these patients. Beyond health education, there have been non-health campaigns that have tackled FGC.

## FGC in law and politics

FGC has been criminalized in 25 African countries (11) – with laws being extended to the developed world, such as the Female Genital Mutilation (FGM) Act in the UK (12). These laws make it illegal to subject a woman to FGC. Despite the obvious support for legal provisions against this practice, there have been flaws with such policies. Many laws are often viewed as ‘symbolic’ in nature. In the UK, FGC laws have led to no successful prosecution to date. In Senegal, the parliamentarian who introduced the new law underlined that the courts would not even apply it in judicial proceedings (13). Many researchers have stated the legal policies alone will not change behaviours that propagate FGC – policy should be met with grass-roots projects with communities in order to achieve long-standing behavioural change. Lone policies have also been found to facilitate the ‘underground’ practice of FGC (14).

Despite failures in domestic policy and societal awareness, there are opportunities to advocate for FGC globally. Despite various United Nations–supported gatherings relating to FGC (14), this health burden is not prioritized within the realm of global health. There is opportunity for this to change, however. In September 2015, the United Nations released its Sustainable Development Goals (SDG) with goal 5 calling for gender inequality (15). Sociologist Jeremy Shiffman argues that policymakers are more likely to prioritise global health issues if they align with their interests (16). In this way, framing FGC as a campaign to tackle SDG goal 5 may increase political and financial interest in funding FGC projects.

Overall, global society has aimed to tackle FGC in multiple ways – with some gaining more successes than others. Despite the current problems revolving around poor societal awareness, ineffective legal proceedings, or poor political backing, there are many lessons we can learn to improve our support mechanisms for FGC patients worldwide. More specifically, stronger political backing, which often equates with increased financial funding, will enable us to support the broader causes of FGC by focusing on behavioural change in communities, and structural and economic barriers within society. With these structures put in place, additional health programmes and supportive policies will enable us to tackle FGC both domestically and globally – with the ultimate aim of removing FGC as a global health burden affecting the most vulnerable girls and women worldwide.

*Nina Al-Saadi* Imperial College London London, United Kingdom Email: na2811@ic.ac.uk *Harun Khan* Imperial College London London, United Kingdom *Sameera Auckburally* Imperial College London London, United Kingdom *Adam Al-Saadi* St George's University of London London, United Kingdom *Tasnia Khan* Imperial College London London, United Kingdom
